# The NPR1-dependent salicylic acid signalling pathway is pivotal for enhanced salt and oxidative stress tolerance in *Arabidopsis*

**DOI:** 10.1093/jxb/eru528

**Published:** 2015-01-22

**Authors:** Maheswari Jayakannan, Jayakumar Bose, Olga Babourina, Sergey Shabala, Amandine Massart, Charlotte Poschenrieder, Zed Rengel

**Affiliations:** ^1^School of Earth and Environment, Faculty of Science, University of Western Australia, Crawley WA 6009, Australia; ^2^School of Land and Food, University of Tasmania, Hobart TAS 7001, Australia; ^3^School of Biological Sciences, University of Tasmania, Hobart TAS 7001, Australia; ^4^Fisiología Vegetal, Facultad de Biociencias, Universidad Autónoma de Barcelona, E-08193 Bellaterra, Spain; ^5^Environmental Science and Engineering Section, Ecole Polytechnique Fédérale de Lausanne, CH 1015 Lausanne, Switzerland

**Keywords:** ROS, membrane potential, oxidative stress, potassium fluxes, proton fluxes, salinity, salicylic acid, sodium fluxes, viability staining.

## Abstract

The role of endogenous salicylic acid (SA) signalling cascades in plant responses to salt and oxidative stresses is unclear. *Arabidopsis* SA signalling mutants, namely *npr1-5* (*non-expresser of pathogenesis related gene1*), which lacks NPR1-dependent SA signalling, and *nudt7* (*nudix hydrolase7*), which has both constitutively expressed NPR1-dependent and NPR1-independent SA signalling pathways, were compared with the wild type (Col-0) during salt or oxidative stresses. Growth and viability staining showed that, compared with wild type, the *npr1-5* mutant was sensitive to either salt or oxidative stress, whereas the *nudt7* mutant was tolerant. Acute salt stress caused the strongest membrane potential depolarization, highest sodium and proton influx, and potassium loss from *npr1-5* roots in comparison with the wild type and *nudt7* mutant. Though salt stress-induced hydrogen peroxide production was lowest in the *npr1-5* mutant, the reactive oxygen species (ROS) stress (induced by 1mM of hydroxyl-radical-generating copper-ascorbate mix, or either 1 or 10mM hydrogen peroxide) caused a higher potassium loss from the roots of the *npr1-5* mutant than the wild type and *nudt7* mutant. Long-term salt exposure resulted in the highest sodium and the lowest potassium concentration in the shoots of *npr1-5* mutant in comparison with the wild type and *nudt7* mutant. The above results demonstrate that NPR1-dependent SA signalling is pivotal to (i) controlling Na^+^ entry into the root tissue and its subsequent long-distance transport into the shoot, and (ii) preventing a potassium loss through depolarization-activated outward-rectifying potassium and ROS-activated non-selective cation channels. In conclusion, NPR1-dependent SA signalling is central to the salt and oxidative stress tolerance in *Arabidopsis*.

## Summary

NPR1-dependent salicylic acid signalling controls sodium entry into the roots while preventing potassium loss through depolarization-activated outward-rectifying potassium and ROS-activated non-selective cation channels during salt and oxidative stresses.

## Introduction

Soil salinity is one of the major abiotic stresses that threaten sustainable food production worldwide. About 831 million ha of land is affected by natural salinization worldwide (Rengasamy, 2006). In addition, secondary salinization, resulting from poor irrigation and/or drainage practices, affects more than 50% of productive irrigated land globally (Martinez-Beltran and Manzur, 2005), increasing on average by up to 0.5M ha each year. Remediation of salt-affected arable lands is very expensive, time consuming, and hard to implement on a large scale. Thus, increasing the salt tolerance of crop plants through molecular and plant breeding approaches is the most attractive and viable option to sustain food production in salt-affected environments (Ondrasek *et al.*, 2011). In this regard, salicylic acid (SA) has gained importance as an important signalling phytohormone that can marshal salt tolerance in plants (Borsani *et al.*, 2001; Horváth *et al.*, 2007). However, the exact SA signalling cascades during salt stress remain elusive.

Endogenous SA is synthesised from a primary metabolite, chorismate, by two distinct pathways: the phenylalanine ammonia-lyase pathway in the cytoplasm, and the isochorismate pathway in the chloroplast (reviewed in Dempsey *et al.*, 2011; Rivas-San Vicente and Plasencia, 2011). The latter pathway is responsible for the bulk of the pathogen-induced SA synthesis in diverse plant species (reviewed in Vlot *et al.*, 2009). An *Arabidopsis sid2* (*SA induction deficient 2*) mutant defective in the expression of the isochorismate synthase (*ICS1*) gene is hypersensitive to salt stress (Lee *et al.*, 2010; Asensi-Fabado and Munné-Bosch, 2011), implying that this pathway is essential for salinity tolerance in plants. In contrast, some studies have found that a SA-deficient *Arabidopsis* mutant exhibited higher salinity stress tolerance compared with the wild type and SA-hyper-accumulating mutants (Borsani *et al.*, 2001; Cao *et al.*, 2009; Hao *et al.*, 2012). However, opposite to the aforementioned results were also reported by some other authors (Asensi-Fabado and Munné-Bosch, 2011; Miura *et al.*, 2011). The reason for such discrepancy is due to the use of mutants that were not altered in the isochorismate-synthase-mediated SA synthesis causing subsequent changes in SA accumulation. Instead, the SA levels were altered by SA hydroxylase (*NahG*) activity, allowing for the possibility that SA signalling might be turned on before *NahG* converts SA into catechol (Borsani *et al.*, 2001). Moreover, among the SA biosynthesis pathways, only the isochorismate-synthase-mediated SA synthesis pathway is stress inducible (see above); hence, it is imperative to evaluate specifically the isochorismate-synthase-mediated SA-hyper-accumulating mutants during salt stress to decipher SA signalling.

The *Arabidopsis* genome contains 25–32 Nudix (nucleoside diphosphates linked to moiety X) hydrolases (AtNUDTs) that hydrolyse nucleoside derivatives (Kraszewska, 2008); however, the work on estimating the number of Nudix genes is ongoing. Among the members, AtNUDT7 (At4g12720) was identified as a gene induced by multiple stresses, including salinity (Jambunathan and Mahalingam, 2006), and its knockout mutant, *nudt7-1* (SALK_046441; formerly known as *growth factor gene 1*; hereafter described as *nudt7*) was found to have three- to four-fold higher concentration of SA than the wild type under control growth conditions (Bartsch *et al.*, 2006; Straus *et al.*, 2010; Wang *et al.*, 2012). This SA concentration increase is absent in the double mutant *nudt7 sid2-1* (Bartsch *et al.*, 2006; Straus *et al.*, 2010), suggesting that isochorismate-synthase-mediated SA biosynthesis is responsible for high SA in *nudt7* mutant. Hence, characterization of *nudt7* mutant under salt stress may be a useful tool to answer whether isochorismate-synthase-mediated SA biosynthesis and SA accumulation are essential for salt tolerance in plants.

To activate a defence response, SA should bind to some specific receptors. The NPR1 (non-expresser of pathogenesis-related gene 1) protein was identified as one of these (Wu *et al.*, 2012). Simultaneous studies revealed that SA also binds with NPR1 prologues NPR3 and NPR4, which in turn trigger the reduction of inactive oligomeric NPR1 into active monomeric NPR1 (a master regulator of SA-induced defence genes) in the cytoplasm (Fu *et al.*, 2012). The monomeric NPR1 enters the nucleus and functions as a transcriptional co-activator of defence genes (Attaran and He, 2012; Fu *et al.*, 2012). Microarray analysis in *Arabidopsis* reported that among SA-induced defence genes, more than 90 percent were NPR1-dependent genes (Wang *et al.*, 2006; Blanco *et al.*, 2009). In particular, the *Atnudt7* mutant has been reported to mediate both NPR1-dependent and NPR1-independent defence response against pathogens (Ge *et al.*, 2007). Moreover, defence genes that control programmed cell death and osmotic and oxidative stress tolerance (all important for salt tolerance) fall under either pathway (Blanco *et al.*, 2009).

Recently, an *Arabidopsis* NPR1 knockout mutant (*npr1-1*) accumulated SA upon salt stress and showed enhanced salt tolerance (Hao *et al.*, 2012). On the other hand, an NPR1-hyper-accumulating *Arabidopsis* double mutant (*npr3npr4*) failed to undergo programmed cell death (Attaran and He, 2012; Fu *et al.*, 2012), suggesting NPR1-mediated prevention of programmed cell death may be beneficial for salt tolerance. Overall, it seems that salt tolerance in plants can be controlled by both NPR1-independent and NPR1-dependent mechanisms. Comparison of a *nudt7* mutant (which has both constitutively expressed NPR1-independent and NPR1-dependent SA-mediated pathways) with a NPR1 knockout mutant (without SA-mediated NPR1-dependent pathway) will pave the way for characterizing a SA-mediated defence response against salt stress.

Salt stress increases the production of various forms of reactive oxygen species (ROS) namely superoxide (O_2_^**−**^), singlet oxygen (^1^O_2_), hydrogen peroxide (H_2_O_2_), and hydroxyl radical (^.^OH) in plants (reviewed in Parida and Das, 2005). Some of these ROS species (^.^OH, O_2_^**−**^, and H_2_O_2_) can induce K^+^ loss via ROS-activated channels and trigger programmed cell death during salt stress (e.g Shabala *et al.*, 2007; Demidchik *et al.*, 2010; Poór *et al.*, 2012; Tran *et al.*, 2013). Several independent studies confirmed that *Atnudt7* mutant participated in redox homeostasis maintenance (Ge *et al.*, 2007; Ishikawa *et al.*, 2009; Jambunathan *et al.*, 2010; Straus *et al.*, 2010) and delayed programmed cell death (Straus *et al.*, 2010). However, it needs to be tested whether delayed programmed cell death in the *nudt7* mutant is due to prevention of K^+^ loss through ROS-activated channels. Exploring this issue was one of the aims of this study.

The present study hypothesized that the elevated SA concentration may mediate adaptive responses against salt and oxidative stresses through both NPR1-independent and NPR1-dependent pathways. This hypothesis was tested by characterizing roots of *Arabidopsis* mutants, namely *nudt7*, and *npr1-5* under saline and oxidative stresses. The *nudt7* contains the constitutively expressed SA-mediated NPR1-independent and NPR1-dependent defence genes, whereas *npr1-5* (formerly known as *sai1*, salicylic acid-insensitive1), is a NPR1-knockout mutant without the SA-mediated NPR1-dependent defence response (Shah *et al.*, 1997; Shah *et al.*, 1999). The reported results confirm that SA-mediated salt and oxidative stress tolerance is NPR1-dependent. Particularly, NPR1-dependent SA signalling helps plants to (i) prevent Na^+^ loading into root tissue and its subsequent transport into shoots, and (ii) retain K^+^ both in the roots and shoots by controlling K^+^ loss through depolarization-activated outward-rectifying K^+^ channels (KOR) and ROS-activated non-selective cation channels (NSCC).

## Materials and methods

### Plant material

Seeds of *Arabidopsis thaliana* L. wild type (Col-0) and mutant seeds of loss-of-function of *NPR1* gene *npr1-5* (Salk_CS3724, Col-0) and *NUDT7* gene *nudt7* (Salk_046441, Col-0) were obtained from the *Arabidopsis* Biological Resource Centre (http://www.Arabidopsis.org/abrc/). *Arabidopsis* seeds were surface sterilized with 1 % v/v sodium hypochlorite (commercial Bleach) plus 0.01 % v/v Triton (wetting agent) for 10min followed by at least three rinses with sterile deionized water.

### Long-term growth experiments

**Fig. 1.**
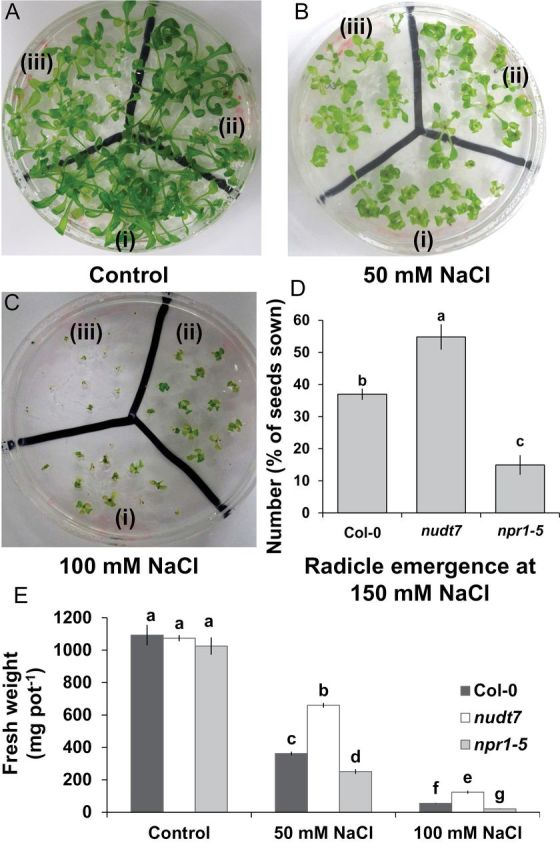
Growth and radicle emergence of *Arabidopsis thaliana* grown in full-strength MS medium with 2% w/v phytogel infused with different concentration of salt. (A–C) Photographs of radicle emergence in (i) Col-0, (ii) *nudt7*, (iii) *npr1-5* at the indicated NaCl concentrations 7 d after sowing. (D) Quantification of radicle emergence out of 20 seeds shown under 150mM NaCl treatment at 7 d after sowing. (E) Fresh weight of the three genotypes under indicated NaCl concentrations 2 weeks after sowing. Each bar in the graphs represents mean±SEM. Different letters in bar graphs indicate significant differences. (This figure is available in colour at *JXB* online.)

For genotype comparison, 15 surface-sterilized seeds of each genotype (Col-0, *nudt7*, and *npr1-5*) were sown on the surface of 90-mm Petri dishes containing solid 0.35 % w/v phytogel, full strength Murashige and Skoog medium (MS; Sigma-Aldrich, Castle Hill, NSW, Australia), 1% w/v sucrose, and various concentrations of NaCl (0, 50, 100, or 150mM). Media pH was adjusted to 5.7 by adding either KOH or HCl. The Petri dishes were divided into three equal parts to accommodate three genotypes per dish (Fig. 1). The Petri dishes containing seeds were sealed with Parafilm strips, kept at 4 °C for 2 d, and then transferred into a growth chamber with 16/8h day/night photoperiod, 150 µmol m^–2^ s^–1^ photon flux density and 23 °C temperature. The Petri dishes were placed in a horizontal position, allowing the roots to grow through the phytogel MS media for 25 d. To assess radicle emergence during salt stress, *Arabidopsis* seeds were sown on the MS media containing 150mM NaCl. Seeds were then vernalized (as above), and the germination percentage was assessed after 7 d in the growth chamber. These experiments were repeated at least twice, with four replicates each time.

At the end of the experiment, plants were harvested and thoroughly rinsed with ice-cold 0.5mM CaSO_4_ solution; excess water was removed by blotting shoots with paper towels, and fresh weight was measured immediately. Plants were then dried at 65 °C for 2 d in a Unitherm Dryer (Birmingham, UK) and weighed. Shoot water content (%) was calculated as the difference between fresh and dry weight.

### Short-term experiments

Surface-sterilized seeds were sown on the surface of 90-mm Petri dishes containing 0.4 % w/v agar, 1.0mM KCl plus 0.1mM CaCl_2_ at pH 5.7 (Jayakannan *et al.*, 2011; Jayakannan *et al.*, 2013). The Petri dishes containing seeds were sealed, vernalized, and grown under controlled conditions as described above. In the short-term experiments, the Petri dishes were placed vertically, allowing the roots to grow down along the agar surface without penetrating it, but being anchored in it via root hairs. The 4- to 5-day-old seedlings were used for all the short-term experiments (measurements of ion fluxes, membrane potential, and root viability).

### Ion flux measurements

The Microelectrode Ion Flux Estimation (MIFE^TM^, University of Tasmania, Hobart, Australia) technique was used to measure net fluxes of H^+^, K^+^, and Na^+^. The principles and methods of this MIFE^TM^ technique can be found in Newman (2001). The details pertinent to microelectrode fabrication, conditioning, and calibration were detailed in previous publications (Jayakannan *et al.*, 2011; Bose *et al.*, 2013; Jayakannan *et al.*, 2013).

### Preparation of *Arabidopsis* seedlings for MIFE measurements

The roots of an intact *Arabidopsis* seedling were immobilized and conditioned in a Petri dish containing 30ml of BSM (basal salt medium; 1mM KCl and 0.1mM CaCl_2_, pH 5.5) for at least 30min before commencing MIFE measurements (Jayakannan *et al.*, 2011; Jayakannan *et al.*, 2013). The Petri dish was then placed on the microscope stage of the MIFE system. Electrodes were positioned at either the distal elongation zone (180–300 µm from the root cap) or mature root zone (>2mm from the root cap) as described in Bose et al. (2010a, b). Ion fluxes were measured under control conditions for 5min before treatment application. Treatments (100mM NaCl; 1mM copper-ascorbate mix; 1 or 10mM hydrogen peroxide) were applied by pipetting the required volume of treatment stock solutions into the bathing solution in the Petri dish. After addition, the bathing solution was thoroughly mixed by sucking into, and expelling from, a pipette approximately five times. The bathing solution was allowed to equilibrate for 1min before recording ion fluxes under treatment conditions; hence, the time required for the stock addition and the establishment of the diffusion gradients is about 40 s (Shabala and Hariadi, 2005). Accordingly, flux measurements during the first minute after treatment applications were discarded from the analysis and appear as gaps in the figures. Transient flux kinetics of K^+^, H^+^, and Na^+^ were measured for specified times.

### Membrane potential measurements

The roots of an intact *Arabidopsis* seedling were gently secured in a measuring chamber in a horizontal position using a Parafilm strip and small plastic blocks. The seedling was then placed in a 10-ml Perspex measuring chamber filled with 7ml of BSM and pre-conditioned as described above. The specific details pertinent to microelectrode preparation, impalement into the epidermal cells of mature root zone, and data recording can be found in previous publications (Bose *et al.*, 2013; Jayakannan *et al.*, 2013). Once a stable membrane potential measurement was obtained for 1min, salt treatment (100mM NaCl) was imposed. The transient membrane potential kinetics was recorded up to 30min after treatment commencement. The membrane potential values of eight individual seedlings were averaged for every genotype and treatment combination.

### Viability staining

Root viability was assessed by fluorescein diacetate/propidium iodide double staining method as described in a previous publication (Bose *et al.*, 2014).

### *In vivo* hydrogen peroxide imaging

The H_2_O_2_ imaging of root tissue was done by following the standard procedure adopted in a previous publication (Bose *et al.*, 2014). The 4- to 5-day-old *Arabidopsis* seedlings were treated with 100mM NaCl in BSM background. At 4h and 24h after salt treatment, the roots were washed with 10mM Tris-HCl buffer and incubated in 25 µM 2′,7′-dichlorofluorescein diacetate (DCF-DA, D6883; Sigma) for 30min at 30 °C. Following DCF-DA incubation, the amount of H_2_O_2_ produced in roots was assessed by visualizing fluorescence intensity using a confocal microscope (Leica TCS SP5, Leica Microsystems). The Argon, visible laser power was set at 20%. Given that the H_2_O_2_ fluorescence intensity at 4h was stronger than at 24h time point, two different settings (and, hence, two different sets of controls) were used to resolve the signal. The acousto-optic tuneable filter (AOTF-488) was set at 10 % and 40 %, and the hybrid detector (HyD) gain was set at 19 and 120 for 4-h and 24-h time points, respectively. The software Leica Application Suite Advanced Fluorescence (LAS AF, Leica Microsystems) used to acquire images, and ImageJ (National Institutes of Health) was used to calculate the mean fluorescence intensity.

### Statistical analysis

Data are reported as means±SEM. Statistical significance of mean values was determined using the standard LSD test at *P*≤0.05 level.

## Results

### *nudt7* and *npr1-5* plants differ in salt sensitivity

Similar to a previous report (Bose *et al.*, 2013), 2 weeks of salt stress had a strong effect on plant growth, with fresh mass, dry mass, and water content all declining significantly and in a dose-dependent manner for all three *Arabidopsis* genotypes tested (Fig. 1 and Supplementary Fig. S1). This decline was smallest in *nudt7* plants, followed by the wild type, and then by *npr1-5* (most sensitive to salinity; Fig. 1 and Supplementary Fig. S1). Furthermore, under control conditions (i.e. no salt), the fresh (Fig. 1) and dry mass (Supplementary Fig. S1) were slightly lower in *npr1-5* plants than the wild type and *nudt7*, but the difference was not statistically significant. At 150mM NaCl, salt-sensitive *npr1-5* had fewer radicles emerging than *nudt7* and the wild type (Fig. 1).

### The extent of salt-induced loss of cell viability was more severe in *npr1-5* than *nudt7* roots

**Fig. 2.**
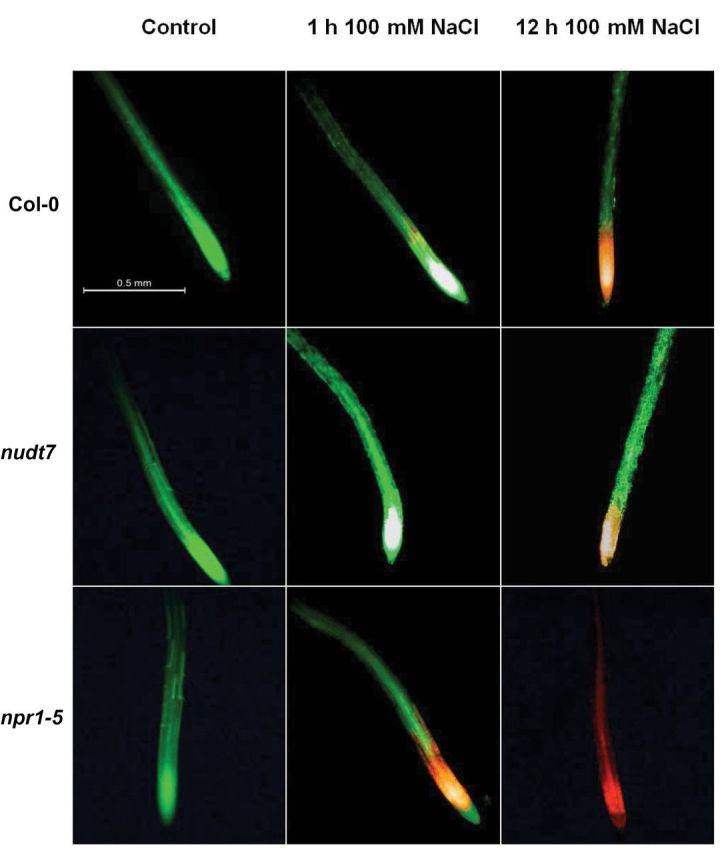
Viability staining images of 4- to 5-day-old *Arabidopsis thaliana* roots exposed to 100mM salt stress. The seedlings were grown in basal salt medium (BSM) containing 0.4% (w/v) agar for 4–5 d, then pre-treated with 100mM NaCl in BSM for 1 or 12h, and double stained with fluorescein diacetate–propidium iodide for imaging under a fluorescence microscope. The control plants were treated only with BSM; the image shown is the control plant after 12h in BSM. (This figure is available in colour at *JXB* online.)

To determine the effect of salinity on root cell viability, 4- to 5-day-old *Arabidopsis* seedlings were exposed to 100mM NaCl for 1 or 12h and then double stained with fluorescein diacetate–propidium iodide (FDA–PI; Fig. 2). Under the fluorescence microscope, viable cells fluoresced bright green, whereas dead/damaged cells fluoresced bright red (Fig. 2). The *Arabidopsis* seedlings incubated in BSM alone (control) showed green fluorescence even after 12h, suggesting the control roots were viable and healthy in our experimental solutions (Fig. 2).

An hour of salt stress severely affected the viability of *npr1-5* root cells in the elongation and meristematic regions, with the wild-type roots also showing a few dead cells in the elongation zone (Fig. 2). However, no damage was observed in the roots of *nudt7* mutant (Fig. 2). Prolonged salt exposure (12h) increased the extent of the damage in the following order *npr1-5* > Col-0 > *nudt7*. These results were consistent with the long-term salinity exposure data (Fig. 1 and Supplementary Fig. S1) and imply that roots of *npr1-5* were sensitive to salt stress, whereas *nudt7* was salt-tolerant.

### NaCl-induced ion flux responses varied between *nudt7* and *npr1-5*

**Fig. 3.**
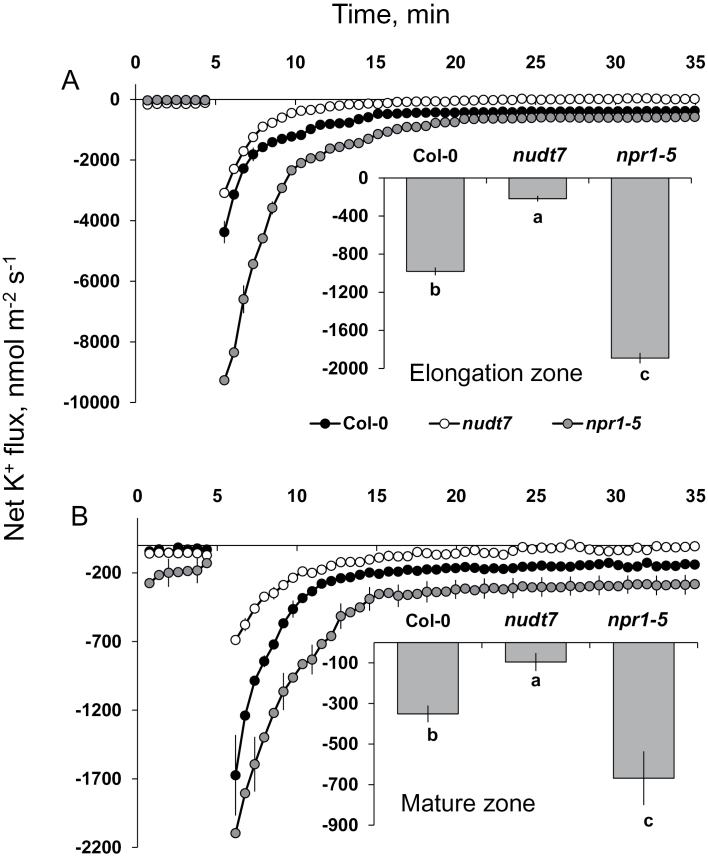
Transient K^+^ fluxes measured at the root elongation and the mature zones of 4- to 5-day-old *Arabidopsis thaliana* seedlings exposed to 100mM salt stress. The insets were average K^+^ fluxes during 1-h exposure to 100mM NaCl stress. Each point or bar represents mean±SEM of 8–12 seedlings. Different letters below the bars in the insets indicate significant differences.

**Fig. 4.**
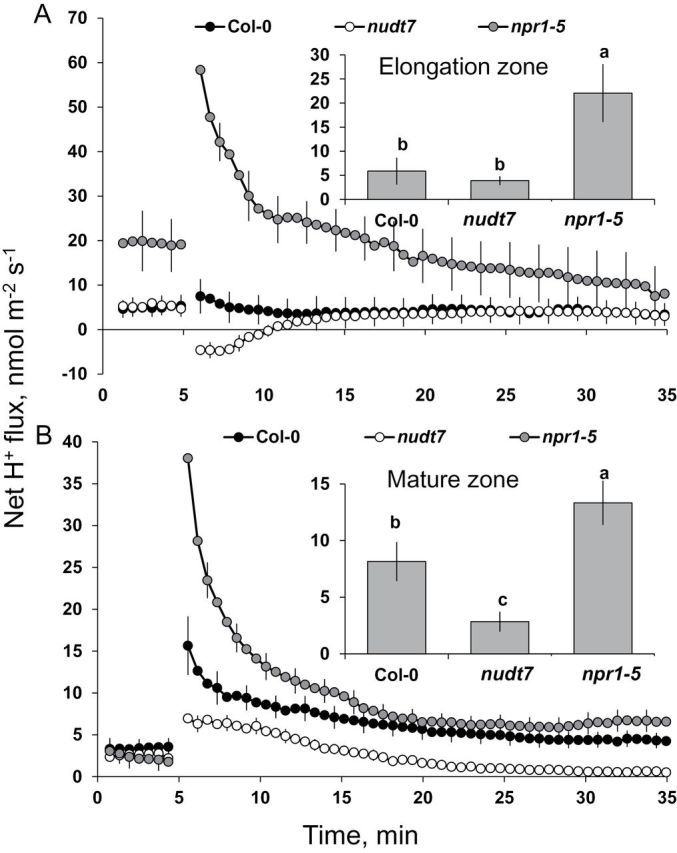
Transient H^+^ fluxes measured at the root elongation and the mature zones of 4- to 5-day-old *Arabidopsis thaliana* seedlings exposed to 100mM salt stress. The insets were average H^+^ fluxes during 1-h exposure to 100mM NaCl stress. Each point or bar represents mean±SEM of 8–12 seedlings. Different letters above the bars in the insets indicate significant differences.

**Fig. 5.**
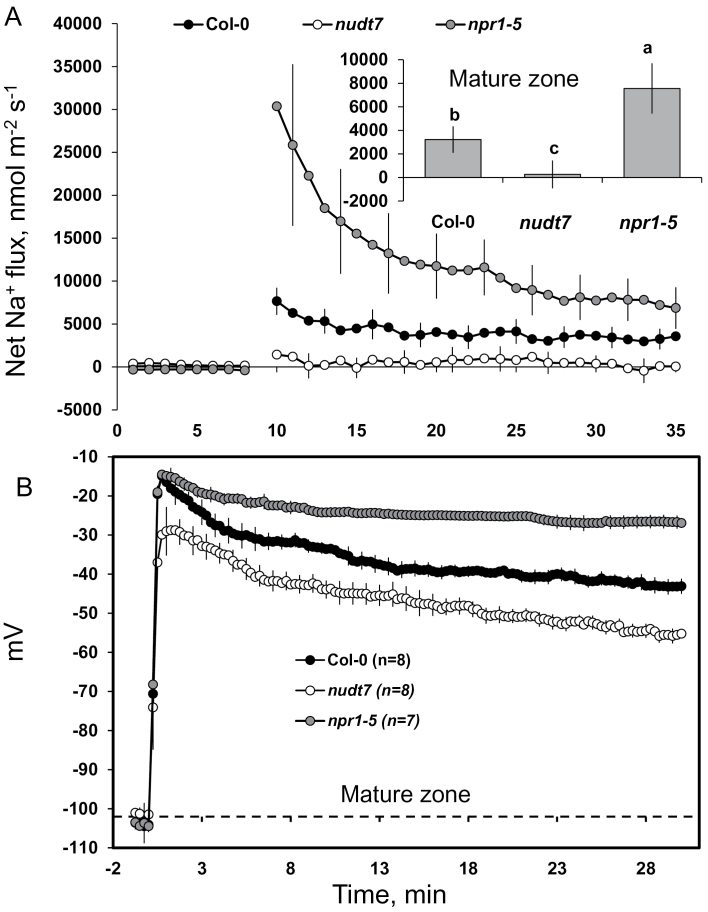
Transient (A) Na^+^ fluxes and (B) membrane potential dynamics measured at the mature root zone of 4- to 5-day-old *Arabidopsis thaliana* seedlings exposed to 100mM salt stress. The inset was average Na^+^ fluxes during 1-h exposure to 100mM NaCl stress. Each point or bar represents mean±SEM of 8–12 seedlings. Different letters above the bars in the inset indicate significant differences.

Consistent with our previous observations on *Arabidopsis* roots (Jayakannan *et al.*, 2011; Bose *et al.*, 2013), salinity (100mM NaCl) caused significant changes in net ion fluxes measured from the elongation and mature zones of *Arabidopsis* roots (Figs 3, 4 and 5).

Acute salt stress caused significant K^+^ efflux from elongation and mature root zones in all genotypes tested (Fig. 3). The peak K^+^ efflux was reached within 2min after imposition of salt stress, followed by gradual recovery and stabilization 20min later. Nearly a 4-fold difference in peak K^+^ fluxes was found between the elongation and the mature root zones in each *Arabidopsis* genotype (Fig. 3), implying the root elongation zone is more sensitive to salt stress than the mature root zone.

Among the three genotypes, the highest NaCl-induced K^+^ efflux was measured from *npr1-5* roots in both the elongation and the mature root zones (–9269±574 and –2096±367 nmol m^–2^ s^–1^, respectively), whereas *nudt7* showed about a 3-fold smaller peak K^+^ efflux (Fig. 3). The wild type had a peak K^+^ efflux in between the two mutants. In addition, the average K^+^ efflux over the first 60min of salt treatment was about 9-fold (elongation zone) and 6-fold (mature zone) higher in salt-sensitive *npr1-5* than salt-tolerant *nudt7* mutant (Fig. 3 insets).

Salinity-induced H^+^ fluxes also showed genotypic differences, in both the elongation and mature root zones (Fig. 4). Under control conditions (no salt), a significantly higher net H^+^ influx was observed in the root elongation zone of the *npr1-5* mutant in comparison with Col-0 and the *nudt7* mutant (Fig. 4 top panel). Addition of 100mM NaCl caused a significant increase in net H^+^ influx in the elongation zone of *npr1-5* (58±8.5 nmol m^–2^ s^–1^) and Col-0 (7.4±4.4 nmol m^–2^ s^–1^; Fig. 4 top panel). By contrast, 100mM NaCl addition induced an initial H^+^ efflux in the elongation zone of the *nudt7* mutant followed by recovery towards the steady state before salt treatment (Fig. 4 top panel). In the mature root zone (Fig. 4, bottom panel), NaCl increased H^+^ influx for all three genotypes with the following magnitude *npr1-5* > Col-0 > *nudt7* (Fig. 4, bottom panel). Similarly, the average H^+^ influx (over the first 60min after salt application) at both the elongation and mature root zones was highest in the *npr1-5* mutant followed by Col-0 and was least in *nudt7* (Fig. 4 insets).

Na^+^ fluxes were measured in the mature root zone of the three *Arabidopsis* genotypes (Fig. 5A) using an improved Na^+^-selective resin (Jayakannan *et al.*, 2011). Acute salt stress caused an immediate Na^+^ influx in Col-0 and *npr1-5* (Fig. 5A). The peak Na^+^ influx was observed within minutes of salt addition and declined thereafter, but remained positive (influx) throughout the measurement period in *npr1-5* and the wild type, while hovering around zero in *nudt7* (Fig. 5A). The average Na^+^ flux measured during 1-h salt stress was about 28-fold higher in *npr1-5* than *nudt7* (Fig. 5A inset).

### *nudt7* and *npr1-5* differ in the magnitude of NaCl-induced depolarization of the plasma membrane

The resting membrane potential in the mature zones of *Arabidopsis* roots was not significantly different among the three genotypes under control conditions (Fig. 5B). Adding 100mM NaCl to the bathing medium resulted in highly significant (*P*≤0.01) membrane depolarization in all three *Arabidopsis* genotypes tested. The time-course of membrane potential changes (Fig. 5B) mirrored both Na^+^ (Fig. 5a) and K^+^ flux (Fig. 3) data, with the maximum membrane depolarization observed within minutes of NaCl treatment; approximately at the same time as the peak Na^+^ influx and K^+^ efflux (the magnitude of the former being greater than that of the latter) (Figs 3 and 5). Initial depolarization was followed by a substantial (10–20 mV) membrane repolarization, with the membrane potential reaching new steady-state values in all three *Arabidopsis* genotypes 20–30min after salt application (Fig. 5B). Among the genotypes, the salt-sensitive *npr1-5* showed the highest magnitude of membrane depolarization (to –15±1 mV), whereas salt-tolerant *nudt7* showed the least membrane depolarization (to –30±1 mV) (Fig. 5B). A ≈25mV difference between *nudt7* and *npr1-5* plants was maintained throughout the measurement period (Fig. 5B).

### Salt-induced H_2_O_2_ production was higher in *nudt7* than *npr1-5*

**Fig. 6.**
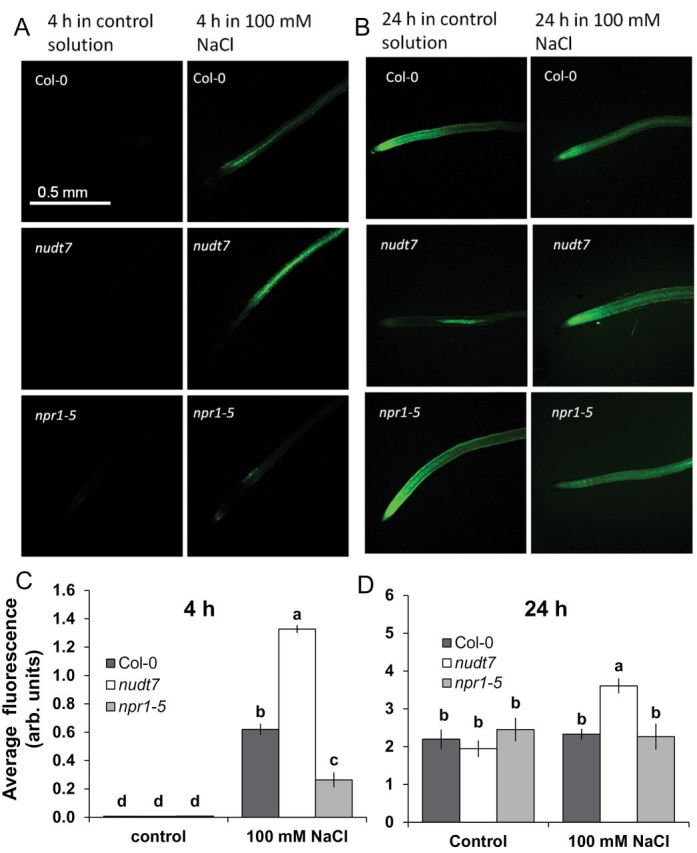
*In vivo* detection of hydrogen peroxide production in the root tissue of *Arabidopsis thaliana* seedlings after NaCl treatment. (A, B) Images of *Arabidopsis thaliana* seedling roots, after being exposed to the indicated salt concentrations for 4 or 24h. Samples were stained with 2′,7′-dichlorofluorescein diacetate for imaging under a fluorescence microscope. Roots for treatments were taken from 4- to 5-day-old seedlings grown in basal salt medium (BSM) containing 0.4% (w/v) agar. Because the hydrogen peroxide fluorescence was much higher at 4h than at 24h, different settings were used to acquire images to show difference between genotypes at each time point. (C, D) Quantification of fluorescence in the roots of the different genotypes after exposure to salt stress for the indicated times. Each bar represents mean±SEM of 8–12 seedlings. Different letters above the bars in the bar graphs indicate significant differences. (This figure is available in colour at JXB online.)

*In vivo* imaging of H_2_O_2_ production in root tissue was done 4h and 24h after 100mM NaCl addition (Fig. 6). The salt-induced H_2_O_2_ production was several folds higher at 4h than 24h in all the genotypes tested, necessitating specific settings (described in the Materials and methods section) to acquire images for each time point to avoid oversaturation and photobleaching. Among the genotypes, mutant *npr1-5* with SA signalling blockage had lower capacity to increase H_2_O_2_ production under salt stress, whereas *nudt7* mutant showed sustained elevation in H_2_O_2_ production under salt stress at both time points.

### Shoot Na and K concentrations differed between *nudt7* and *npr1-5* during long-term salt exposure

**Fig. 7.**
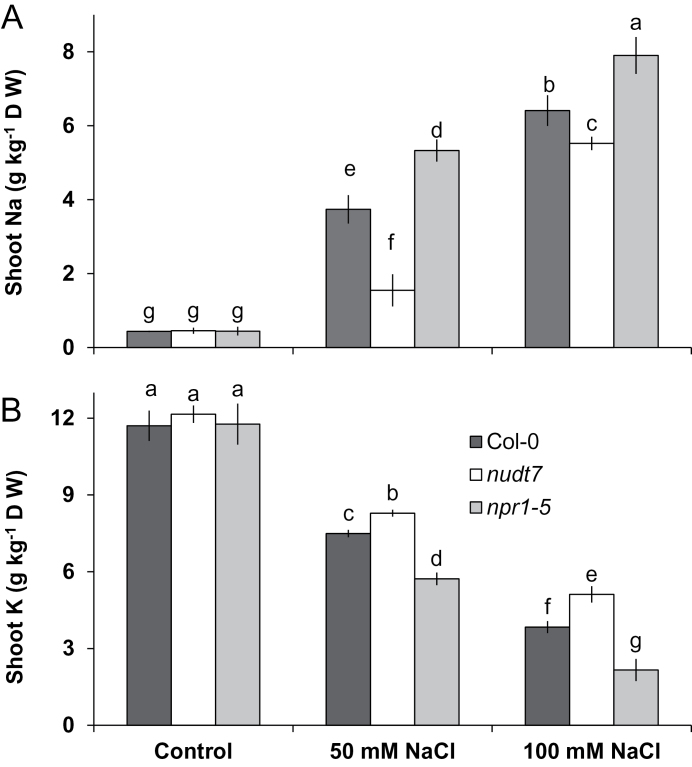
Effect of different NaCl treatment on concentrations of Na^+^ (A) and K^+^ (B) in *Arabidopsis* shoots after 25 d of growth in the full-strength MS medium with 2% w/v phytogel. Each bar represents mean±SEM. Different letters above the bars indicate significant differences.

As expected, 25 d of growth in NaCl-supplemented MS media caused a substantial increase in the shoot Na^+^ concentration and a decrease in the shoot K^+^ concentration in all three *Arabidopsis* genotypes tested (Fig. 7). Under salt stress, *nudt7* showed the lowest Na^+^ concentration in shoots followed by the wild type, whereas the *npr1-*5 mutant had the highest concentration (Fig. 7A). In contrast, the shoot K^+^ concentration was the highest in the *nudt7* mutant followed by the wild type and was lowest in the *npr1-5* mutant (Fig. 7B) under either 50 or 100mM NaCl stress.

### *nudt7* and *npr1-5* mutants vary in their oxidative stress tolerance

**Fig. 8.**
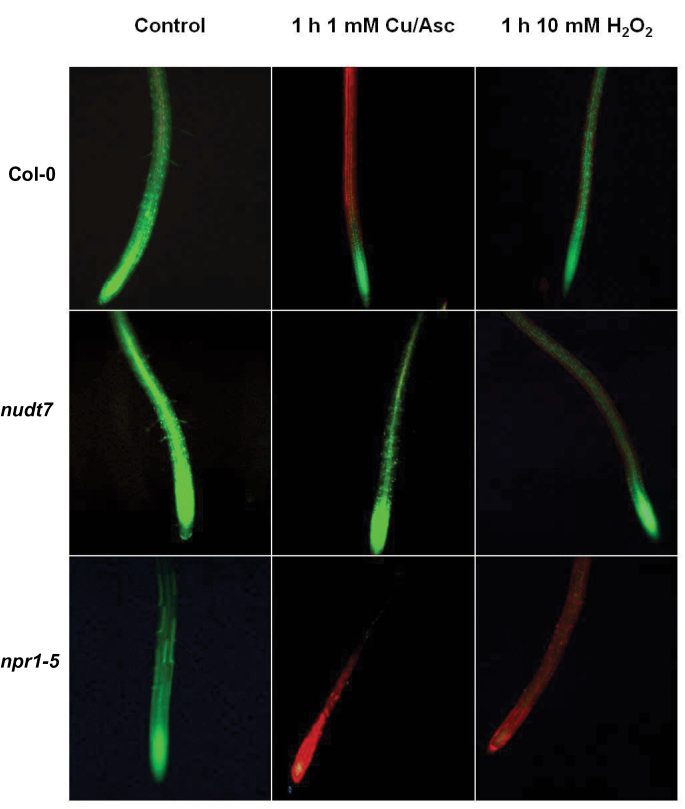
Viability staining of 4- to 5-day-old *Arabidopsis thaliana* roots exposed to 1mM Cu-ascorbate or 10mM H_2_O_2_ for 1h. The seedlings were grown in basal salt medium (BSM) containing 0.4 % w/v agar for 4–5 d, were pre-treated with either 1mM CuCl_2_+1mM ascorbate or 10mM H_2_O_2_ in the BSM background for 1h and then stained with fluorescein diacetate–propidium iodide for observations under a fluorescence microscope. (This figure is available in colour at *JXB* online.)

The viability staining was used to evaluate the responses of *Arabidopsis* genotypes during oxidative stress by treating 4- to 5-day-old seedlings in a hydroxyl-radical-producing medium (1mM copper-ascorbate or 10mM H_2_O_2_) for 1h (Fig. 8). Fluorescence microscopy showed that 1-h exposure to hydroxyl radicals caused severe damage to the roots of *npr1-5* and less so to the wild type Col-0 (Fig. 8). No damage was found in *nudt7* mutant (Fig. 8). Furthermore, in *npr1-5* treated with copper-ascorbate the damage was detected in the root tips as well as in the mature root part, whereas in Col-0 plants only the mature zone showed damage symptoms (Fig. 8). With respect to H_2_O_2_, the damage was smaller in Col-0 and *nudt7* in comparison to *npr1*-5 (Fig. 8). The damage was detected only in the cortex of the mature roots of Col-0 and *nudt7* (Fig. 8), whereas the whole roots were severely affected by H_2_O_2_ stress in *npr1-5*.

### Net ion fluxes influenced by oxidative stress differ between *nudt7* and *npr1-5* mutants

**Fig. 9.**
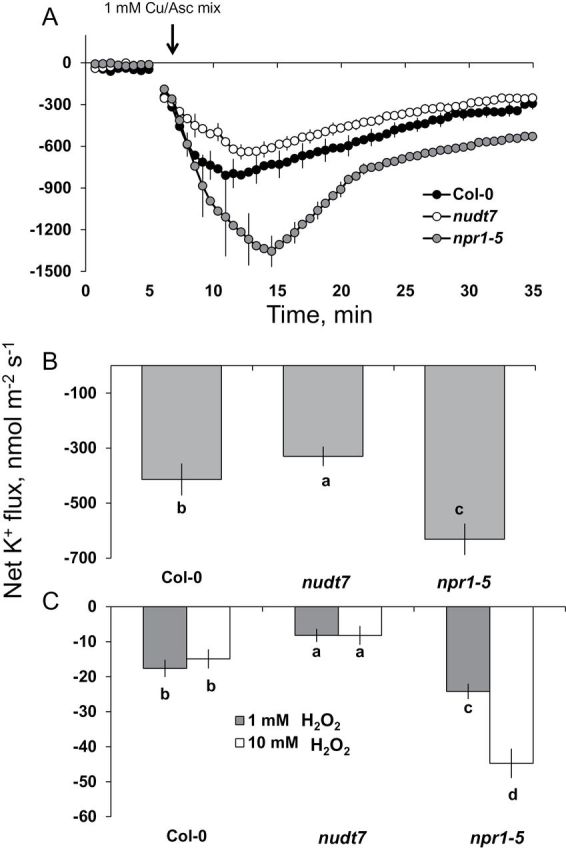
K^+^ fluxes in response to 1mM Cu-ascorbate. (A) Transient K^+^ fluxes in response to 1mM Cu-ascorbate applied after 5min. (B) Average K^+^ fluxes during 1-h exposure to 1mM Cu-ascorbate or (C) 1 or 10mM H_2_O_2_ stress. K^+^ fluxes measured at the mature root zone of 4- to 5-day-old *Arabidopsis thaliana* seedlings. Each point or bar represents mean±SEM of 8–12 seedlings. Different letters below the bars indicate significant differences.

Application of 1mM of hydroxyl-radical-generating copper-ascorbate mix caused a large K^+^ efflux from the mature root zone of all three *Arabidopsis* genotypes (Fig. 9A). This hydroxyl-radical-induced K^+^ efflux was not instantaneous, but increased gradually over time, reaching a peak value 5min after the commencement of the oxidative stress treatment in Col-0 and *nudt7* and 10min for *npr1-5* (Fig. 9A). The magnitude of K^+^ efflux was the lowest in *nudt7* and the highest in *npr1-5* (Fig. 9A; 2-fold difference; significant at *P*≤0.05). The K^+^ flux gradually recovered after reaching the peak, although it remained negative for the treatment duration in all three *Arabidopsis* genotypes (Fig. 9A). The average K^+^ efflux measured over a 60-min Cu-ascorbate treatment period was 2-fold higher in *npr1-5* than *nudt7* (Fig. 9B).

The average K^+^ fluxes during 1-h exposure to either 1 or 10mM H_2_O_2_ treatment revealed no significant dose-dependency in *Arabidopsis* genotypes *nudt7* and Col-0 (Fig. 9C). However, the *npr1-5* mutant had 2-fold greater K^+^ efflux at 10 than at 1mM H_2_O_2_ (Fig. 9C). This mutant had greater K^+^ efflux than *nudt7* and Col-0 regardless of the H_2_O_2_ concentration used (Fig. 9C).

**Fig. 10.**
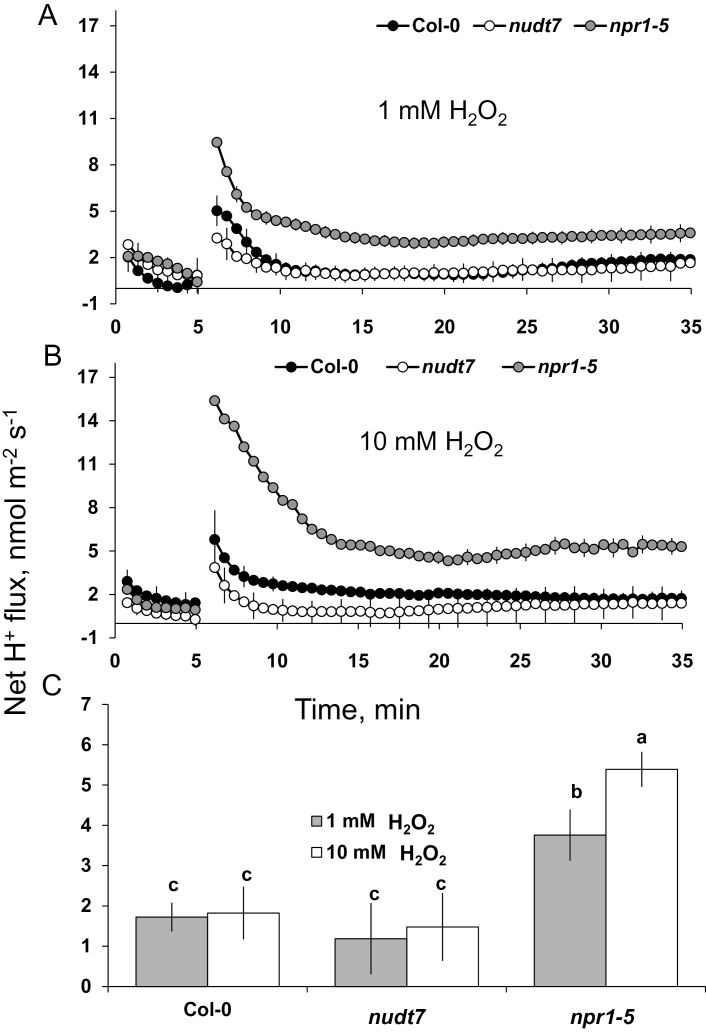
H^+^ fluxes in response to treatment with H_2_O_2_. (A, B) Transient H^+^ fluxes measured at the mature root zone of 4- to 5-day-old *Arabidopsis thaliana* seedlings in response to 1 or 10mM H_2_O_2_. (C) Average H^+^ fluxes during 1-h exposure to 1 or 10mM H_2_O_2_. Each point or bar represents mean±SEM of 8–12 seedlings. Different letters above the bars in bar graph indicate significant differences.

Though the initial H^+^ flux from the mature root zone of *Arabidopsis* was higher at 10mM H_2_O_2_ than 1mM H_2_O_2_, the steady state H^+^ flux (from 10min onwards) is similar for different genotypes exposed to either concentration of H_2_O_2_ (Fig. 10). In general, the salt-sensitive *npr1-5* mutant showed significantly higher (4- to 5-fold) H^+^ influx compared with the other two genotypes (*nudt7* and Col-0) in either 1 or 10mM H_2_O_2_ (Fig. 10).

## Discussion

### The NPR1-dependent SA signalling is pivotal for Na^+^ exclusion from roots and shoots

Maintaining relatively low Na^+^ concentration in shoots is an important trait for salt tolerance in glycophytes (Colmer *et al.*, 2005; Munns and Tester, 2008). The main mechanisms employed by the glycophytes to minimize Na^+^ accumulation in shoots are linked to the enhanced capacity of plants to (i) restrict the initial entry of Na^+^ ions into the root tissue, (ii) excrete Na^+^ from root tissue back into the rhizosphere, (iii) sequester Na^+^ inside the root vacuoles, and (iv) reduce the long-distance transport of Na^+^ into the shoots (Cuin *et al.*, 2011). Given that *Arabidopsis* is a glycophyte, shoot Na^+^ concentration analysis and root Na^+^ flux measurements were employed to ascertain the operation of the above mechanisms in two SA-signalling mutants. The *npr1-5* mutant lacking NPR1-dependent SA-signalling recorded the highest Na^+^ influx into root tissue in comparison with the wild type and *nudt7* mutant (Fig. 5A). If *npr1-5* was efficient in sequestering Na^+^ in root vacuoles or excluding Na^+^ out of root cells, there would have been significant improvement in growth accompanied by reduction in the shoot Na^+^ concentration. However, poor growth (Fig. 1 and Supplementary Fig. S1) and viability of root cells (Fig. 2) along with the highest shoot Na^+^ concentration (Fig. 7A) in comparison with the wild type and *nudt7* mutant implied that the *npr1-5* mutant was defective in preventing the entry of Na^+^ into root tissue and its subsequent transport into the shoots.

In contrast to *npr1-5*, the *nudt7* mutant had the lowest Na^+^ influx into root tissue (Fig. 5a). This may be attributable to either decreased Na^+^ entry or enhanced Na^+^ extrusion via H^+^-ATPase-energized SOS1 (a Na^+^/H^+^ exchanger) activity in the plasma membrane (Cuin *et al.*, 2011). Four lines of evidence favour the latter explanation for the *nudt7* mutant. First, the initial Na^+^ entry into the epidermis of root tissue during acute salt stress is thermodynamically passive and is poorly controlled in glycophytes (Tester and Davenport, 2003). Second, the inherent stability of *SOS1* mRNA is poor (with a half-life of only 10min), and it was shown that exogenous H_2_O_2_ application increased the stability of *SOS1* in a rapid (within 30min) concentration-dependent manner (Chung *et al.*, 2008). If this is the case, sustained elevation of H_2_O_2_ production in the root tissue of *nudt7* mutant (Fig. 6) during salt stress is expected to result in improved *SOS1* mRNA stability. Thirdly, *SOS1* transcripts were found to be higher in roots of the salt-tolerant mutant over-expressing haem oxygenase (EC 1.14.99.3) (Bose *et al.*, 2013). Indeed, a 3-fold higher induction of putative haem oxygenase (At1g69720) was found in the *nudt7* mutant when grown under nutrient stress (Jambunathan *et al.*, 2010). Finally, the *nudt7* mutant showed either H^+^ efflux or reduced net H^+^ influx during acute salt stress (Fig. 4) in comparison with the wild type and *npr1-5* mutant, which is usually the result of enhanced H^+^-ATPase activity fuelling SOS1 operation (Bose *et al.*, 2013; Jayakannan *et al.*, 2013). Overall, the above results suggest that the *nudt7* mutant has enhanced capacity to decrease both the loading of Na^+^ into the root tissue and the transport of Na^+^ into the shoot (Fig. 7A).

### The NPR1-dependent SA signalling assists plants in retaining K^+^ during salt stress by controlling both depolarization-activated KOR and ROS-activated NSCC channels

Salinity stress has ionic, hyperosmotic, and oxidative stress components that severely hamper plant growth and productivity. Apart from hyperosmotic stress, both the ionic stress through depolarization-activated KOR and the oxidative stress through ROS-activated non-selective cation channels (NSCC) exacerbate K^+^ loss, thereby depleting the cytosolic K^+^ pool available for metabolic functions, which eventually leads to cell death (Shabala and Cuin, 2008; Shabala, 2009). Hence, the magnitude of salt-induced K^+^ loss can be used as a measure of salt tolerance of diverse plant species, including *Arabidopsis* (Bose *et al.*, 2013; Jayakannan *et al.*, 2013). Acute salt stress in the study presented here resulted (as expected) in a K^+^ loss from both the elongation and mature root zones of all three genotypes tested (Fig. 3). However, the salt-induced K^+^ loss was lowest in the *nudt7* mutant and highest in the *npr1-5* mutant (Fig. 3), suggesting NPR1-dependent SA signalling is critical for decreasing the K^+^ loss during salt stress.

In *Arabidopsis*, comparison of the depolarization-activated KOR knock-out mutant *gork1-1* with *rbhoD* (a mutant lacking ROS production via NADPH oxidase) during acute 100mM NaCl stress revealed that 3/4 of K^+^ loss were mediated by depolarization-activated KOR and the remaining 1/4 through H_2_O_2_-activated channels (Jayakannan *et al.*, 2013). Superoxide (Tran *et al.*, 2013) and hydroxyl radicals (Demidchik *et al.*, 2010) can also induce K^+^ loss through the GORK channel. Thus, the contrasting capacity of *nudt7* and *npr1-5* mutants to retain K^+^ in roots (Fig. 3) and shoots (Fig. 7B) during salt stress may be underpinned by their differential K^+^ loss through KOR and/or ROS-activated NSCC channels.

The entry of positively charged Na^+^ (Fig. 5A) and H^+^ (Fig. 4) ions into root tissue during acute 100mM NaCl stress resulted in net depolarization of the plasma membrane in all three genotypes tested (Fig. 5b), implying that the bulk of the NaCl-induced K^+^ loss (Fig. 3) might have been through depolarization-activated KOR channels. Among the genotypes, H^+^ and Na^+^ uptake (Figs 4, 5A) as well as NaCl-induced membrane depolarization were highest in the *npr1-5* mutant followed by the wild type, and were lowest in the *nudt7* mutant. Moreover, approximately a 15–25 mV difference was observed between *npr1-5* and *nudt7* mutants (the latter being less depolarized) throughout the measurement period (Fig. 5B). Such a difference in depolarization voltage may be associated with a lower NaCl-induced K^+^ loss in *nudt7* compared with *npr1-5*. It is evident that NPR1-mediated SA signalling plays a key role in regulating the membrane potential during salt stress.

An increase in the production of superoxide (Borsani *et al.*, 2001), hydrogen peroxide (Xie *et al.*, 2011), and hydroxyl radicals (Demidchik *et al.*, 2010) was noted in *Arabidopsis* roots exposed to salt stress. These ROS species can promote K^+^ loss through NSCC channels (Demidchik *et al.*, 2003; Zepeda-Jazo *et al.*, 2011) and/or through KOR channels (Demidchik *et al.*, 2010; Tran *et al.*, 2013). The results here (Fig. 9) showed that hydroxyl radicals caused a severe K^+^ loss (about 15- to 20-fold higher) compared with up to 10mM H_2_O_2_. Among the genotypes, the *npr1-5* mutant showed a higher K^+^ loss than the wild type and *nudt7* mutant under hydroxyl radical and hydrogen peroxide treatments (Fig. 9), suggesting *npr1-5* was more sensitive to these ROS species in comparison with the wild type and *nudt7* mutant. The viability staining results confirmed this, whereby a 1-h treatment with either hydroxyl radicals or 10mM hydrogen peroxide affected root cells more severely in *npr1-5* than in the *nudt7* mutant (Fig. 8). The *nudt7* mutant was able to increase the salt-induced H_2_O_2_ production in root tissue over a 24h period, but the *npr1-5* mutant was not (Fig. 6) suggesting NPR1 is a key regulator of salt-induced H_2_O_2_ production in plants. Because the *nudt7* mutant produced more ROS than wild type and *npr1-5* during salt stress, it is reasonable to assume that H_2_O_2_-induced K^+^ efflux would be greater in *nudt7*. However, in the exogenous H_2_O_2_ treatment (1 and 10mM), the K^+^ efflux of *nudt7* mutant did not differ from the wild type, and was lower than in the *npr1-5* mutant (Fig. 9C). This suggests that the presence of an NPR1-mediated SA signalling component in the *nudt7* mutant makes K^+^-efflux transporters insensitive to elevated H_2_O_2_ concentration during salt stress. Overall, these results provide evidence that (i) NPR1-mediated SA signalling is pivotal for H_2_O_2_ production during salt stress, and also for decreasing K^+^ loss through the NSCC and KOR channels activated by hydrogen peroxide and hydroxyl radicals, and (ii) the *nudt7* mutant shows no response to hydrogen peroxide and is tolerant to hydroxyl radicals.

In summary, an *npr1-5* mutant lacking the NPR1-dependent SA signalling was unable to control both the entry of Na^+^ into roots and its long-distance transport into the shoot, and to prevent K^+^ loss via depolarization-activated KOR and the ROS-activated NSCC channels during salt stress. As a result, the *npr1-5* mutant was sensitive to salt stress. On the other hand, the constitutive expression of NPR1-dependent SA signalling enhanced the salt tolerance of a *nudt7* mutant by controlling Na^+^ entry into the root tissue and subsequent transport to the shoot, as well as minimizing K^+^ loss during salt stress. In conclusion, NPR1-dependent SA signalling is a crucial component of salt and oxidative stress tolerance in *Arabidopsis*.

## Supplementary data

Supplementary data are available at *JXB* online

Figure S1. Effect of salt stress on dry weight and water content of *Arabidopsis thaliana* seedlings grown in the full-strength MS medium with 2% w/v phytogel for two weeks.

Supplementary Data

## Abbreviations:

AtNUDT: *Arabidopsis* Nudix hydrolase
NPR1: non-expresser of pathogenesis related gene1
SA: salicylic acid.
