# Supplementary material for: The NPR1-dependent salicylic acid signalling pathway is pivotal for enhanced salt and oxidative stress tolerance in Arabidopsis
Source: J Exp Bot. 2015 Jan 22;66(7):1865–75. doi: 10.1093/jxb/eru528 (PMC4378626; doi:10.1093/jxb/eru528)
Supplement: Supplementary Data [file supp_66_7_1865__index.html]

The NPR1-dependent salicylic acid signalling pathway is pivotal for enhanced salt and oxidative stress tolerance in Arabidopsis — The NPR1-dependent salicylic acid signalling pathway is pivotal for enhanced salt and oxidative stress tolerance in Arabidopsis — Supplementary Data 

# The NPR1-dependent salicylic acid signalling pathway is pivotal for enhanced salt and oxidative stress tolerance in *Arabidopsis*

## Supplementary Data

Data files

**Files in this Data Supplement:**

- Supplementary Data - Supplementary Data
