# Supplementary figures and images for: The NPR1-dependent salicylic acid signalling pathway is pivotal for enhanced salt and oxidative stress tolerance in Arabidopsis
Source: J Exp Bot. 2015 Jan 22;66(7):1865–75. doi: 10.1093/jxb/eru528 (PMC4378626; doi:10.1093/jxb/eru528)

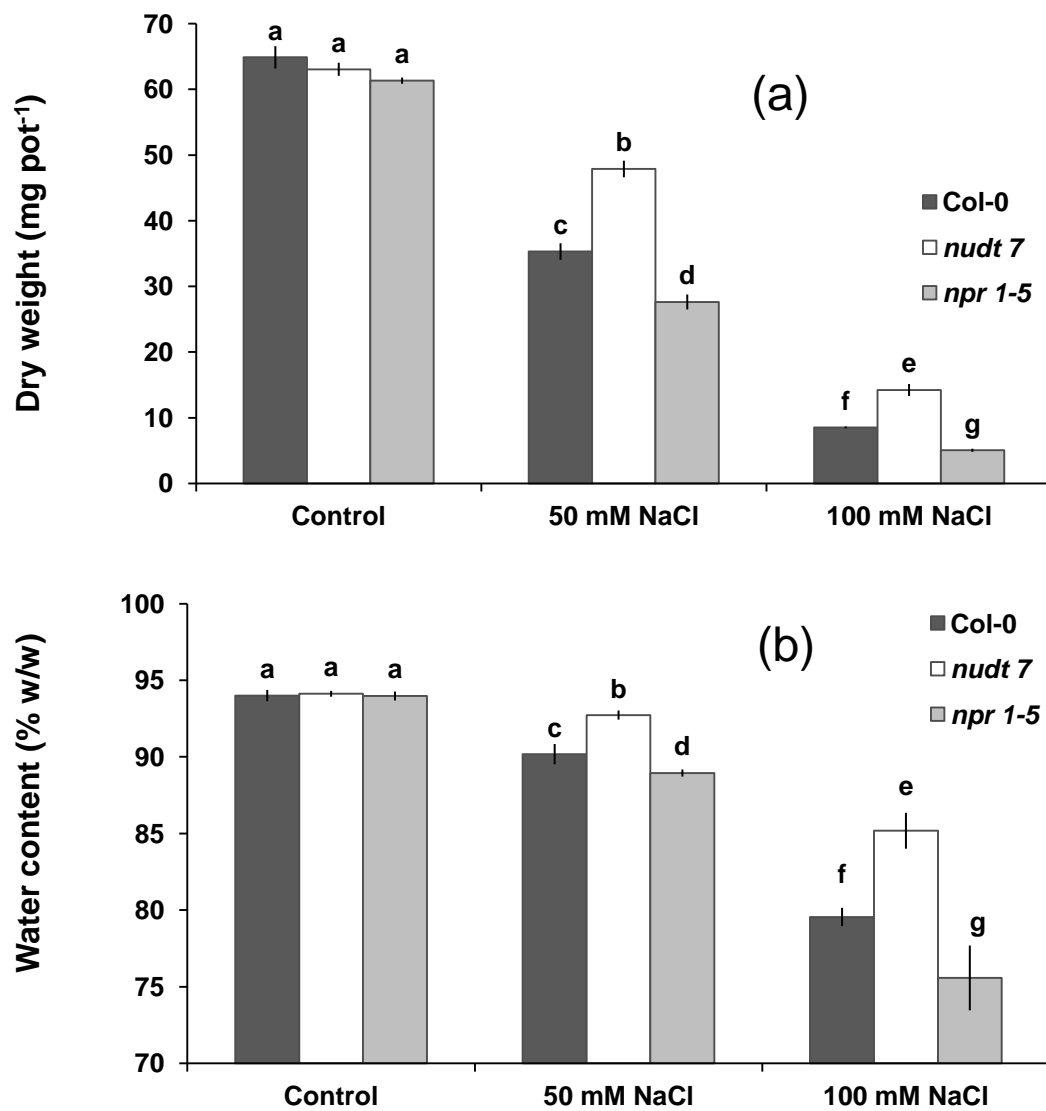

Figure S1.

Supplement: Supplementary Data [file supp_eru528_jexbot133132_file001.pdf]
